# Robotic‐Assisted CT‐Guided Synchronous Percutaneous Lung Biopsy and Cryoablation for Pulmonary Sub‐Solid Nodule: A Case Report

**DOI:** 10.1002/rcr2.70436

**Published:** 2026-04-08

**Authors:** Xiuping Wu, Jielong Lin, Xin Wei, Lianyue Yang, Haoran Xu, Yanwei Chen, Shi Yue Li

**Affiliations:** ^1^ State Key Laboratory of Respiratory Diseases, National Clinical Research Center for Respiratory Disease Guangzhou Institute of Respiratory Health, the First Affiliated Hospital of Guangzhou Medical University Guangzhou China

**Keywords:** biopsy, cryoablation, CT‐guided, ground‐glass nodules, percutaneous, robotic‐assisted

## Abstract

Percutaneous lung biopsy and cryoablation (CRA) under imaging guidance are safe and effective options in the diagnosis and treatment of pulmonary ground‐glass nodules (GGNs) with favourable outcomes. However, percutaneous puncture procedures conventionally require multiple freehand needle manipulations until a satisfactory needle position is achieved, which leads to unnecessary radiation exposure, complications and failure. Despite increasing utilisation of robotic‐assisted systems in recent years, literature describing their use in CT‐guided percutaneous lung biopsy and CRA for pulmonary GGNs remains limited. Herein, we report our initial experience with robotic‐assisted CT‐guided synchronous percutaneous lung biopsy and CRA of a mixed GGN that was accidentally discovered during a physical health examination in a 77‐year‐old female. The 14‐mm‐sized mixed GGN involving the anterior segment and apical segment of the right upper lobe was successfully biopsied and cryoablated without recurrence at the 7‐month follow‐up. This case highlights the feasibility, safety and greater accuracy of employing a novel robotic‐assisted system in lung biopsy and CRA for pulmonary sub‐solid nodules.

## Graphical abstract

This case report demonstrates the successful application of a novel robotic‐assisted system for percutaneous lung biopsy and cryoablation of a pulmonary sub‐solid nodule. The technology enabled precise 3D trajectory planning and accurate needle placement, resulting in complete ablation with no recurrence at 7‐month follow‐up. Robotic assistance presents a promising advancement for improving the precision and outcomes of CT‐guided lung biopsy and ablation procedures.

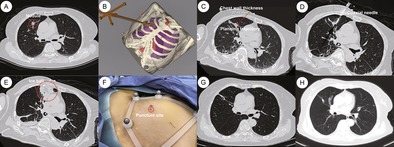

## 1 Introduction

Ground‐glass nodules (GGNs), characterised by ground‐glass opacity and commonly linked to precancerous lesions or early‐stage lung adenocarcinoma, constitute approximately 20%–40% of pulmonary nodules detected in China among patients undergoing low‐dose computed tomography (LDCT) screening [1]. CT‐guided percutaneous lung biopsy is an established, minimally invasive approach for tissue acquisition from pulmonary nodules for pathologic diagnosis. Surgical resection is currently the standard of care for early‐stage lung cancer treatment. Considering the poor cardiopulmonary function, high‐risk comorbidities for surgery and severe surgical anxiety, some individuals may not be suitable surgical candidates. Cryoablation (CRA) may be considered as an alternative option, with the advantage of simultaneously combining with other treatment strategies.

CRA is increasingly being used for suspected malignant nodule management, due to its safety, effectiveness, feasibility, minimal procedural pain and immunological effect [2, 3]. However, manual ablation to the target solely by the operator presents significant challenges due to factors including the reliance on experience, the precision required and the complexity of human anatomy. Some robotic‐assisted systems have been developed in recent years to assist with tumour ablation, which adequately meet the clinical need and achieve satisfactory surgical outcomes [4, 5].

To our knowledge, lung biopsy and CRA of pulmonary sub‐solid nodules with the assistance of a robotic‐assisted system have not been reported in the literature. We report the first and successful treatment of an elderly woman with a 14‐mm‐sized mixed GGN, using robotic‐assisted CT‐guided percutaneous CRA.

## 2 Case Report

A 77‐year‐old female presented to our hospital after an abnormal chest radiography was incidentally detected during a routine health examination. A 14‐mm‐sized mixed GGN in the anterior segment and apical segment of the right upper lobe, exhibiting spiculation, lobulation and vascular encasement, was identified and found to be hypermetabolic on positron emission tomography–computed tomography performed at another hospital, raising suspicion for malignancy. Medical history was notable for hypertension managed with amlodipine, asthma and prior hysterectomy for cervical cancer. Pulmonary function evaluation revealed small‐airway dysfunction (maximal mid‐expiratory flow, 37.9% of predicted) and a paradoxical response to bronchodilator challenge (pre‐bronchodilator: 1.52L, post‐bronchodilator: 1.73L). Based on the aforementioned preliminary diagnoses, three therapy regimens including direct surgical resection with postoperative pathological diagnosis, biopsy followed by personalised comprehensive treatment with a definite pathological diagnosis, and concurrent biopsy plus ablation, were discussed by Multi‐Disciplinary Treatment (MDT). Considering the age, pulmonary function and surgical anxiety, robotic‐assisted percutaneous lung biopsy and CRA were chosen as a treatment strategy after shared decision‐making.

**FIGURE 1:**
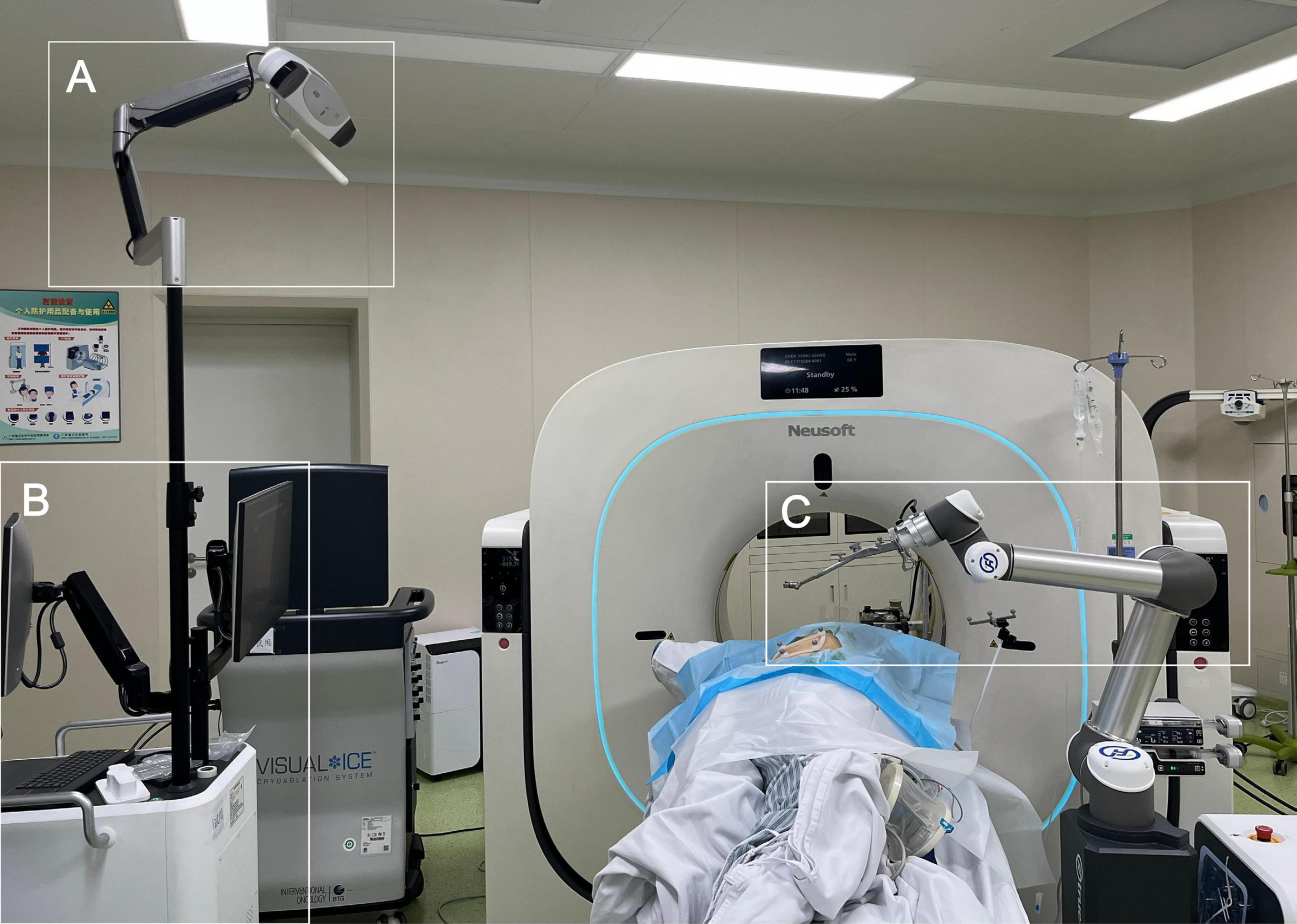
(A–C) The robotic‐assisted navigation system. (A) The photoelectric navigation system allowed for precise tracking of the position of the patient and the robotic arm. (B) The surgical planning system was used to plan the needle insertion trajectory. (C) The robotic arm positioning and puncture system was employed for stable needle‐holding and guiding needle insertion.

**FIGURE 2:**
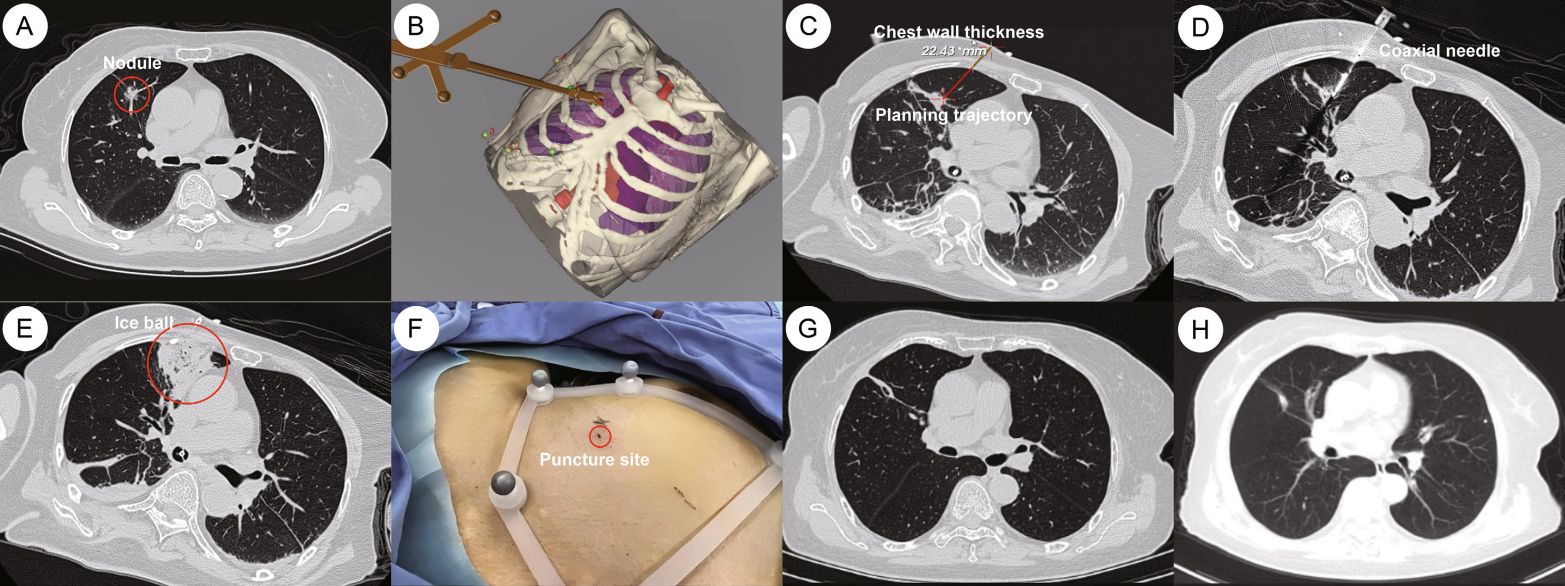
(A–H) Follow‐up and robotic‐assisted CRA procedural images of a 77‐year‐old woman diagnosed with a pulmonary sub‐solid nodule. (A) Initial CT scan image, showing a 14‐mm‐sized mixed GGN involving the anterior segment and apical segment of the right upper lobe (red circle). (B) 3D model reconstruction of the thoracic structure. (C) Planning of the needle trajectory (red line) based on the initial CT scan. (D) Intra‐procedure imaging, showing coaxial needle inside the lesion during ablation procedure. (E) Post‐procedure imaging, showing an ice‐ball of sufficient size (red circle) completely ablates the nodule. (F) Minimal trauma with only a small entry point (red circle) on the skin of our patient (black mark indicated a comparison with conventional free‐hand manipulation). (G) Three‐month follow‐up imaging, showing a scar formation in the ablation zone. (H) Seven‐month follow‐up imaging, showing a successful ablation with no recurrence. 3D = three‐dimensional, CRA = cryoablation.

Robotic‐assisted CT‐guided percutaneous lung biopsy and CRA were finally performed in the supine position using a National Medical Products Administration approved robotic‐assisted system (TH‐S1, TrueHealth Medical Technology Co. Ltd., Hengqin, China) (Figure 1A–C). After importing the preoperative CT images into the robotic‐assisted system, a three‐dimensional (3D) model of the patient was reconstructed and subsequently registered together with the positional information (Figure 2A,B). Based on the 3D model, the trajectory for puncture was determined by the operators via the user interface and then translated into the physical surgical environment (Figure 2C). The robotic arm automatically navigated to the target region along a predetermined trajectory with stable positional and angular control, facilitating single‐pass coaxial needle placement that obviated intra‐procedural repositioning and consequently reduced the number of required CT scans (Figure 2D). Through the coaxial needle, the biopsy yielded two core specimens, which demonstrated inflammation on postoperative histopathologic examination. A 17‐G cryoprobe with a gas‐based cryoablation system using argon and helium (Boston Scientific, Marlborough, MA, USA) was subsequently advanced through the coaxial needle along the established trajectory to the anterior margin of the nodule. A three‐phase CRA protocol was utilised, which consisted of 5‐min, 10‐min and 10‐min freezes, followed by 3‐min thawing cycles. After completion of the three‐phase protocol, the probe was manually repositioned to the posterior margin of the nodule along the same trajectory to repeat the three‐phase protocol and ensure complete coverage of the lesion. The procedure was performed under general anaesthesia, with a total duration of 11 min from the initial CT scan to coaxial needle insertion to the target position, and 83 min for the robotic‐assisted lung biopsy and CRA procedure. An ice ball with sufficient size covered the lesion completely at the time of the immediate postprocedural CT scan with minor pneumothorax (Figure 2E). Despite the occurrence of pneumothorax, the trauma related to the CRA was minimal, and our patient was subsequently discharged home with a quick recovery (Figure 2F). Despite the development of a minor pneumothorax, CRA‐related trauma was minimal; the patient recovered rapidly with oxygen therapy and was discharged 2 days later. Follow‐up imaging conducted 3 months after the procedure showed obvious postoperative changes in the right upper lobe, including local scar band formation, indicating that the tumour cells had been inactivated (Figure 2G). The mixed GGN was judged to have been successfully ablated, with no recurrence observed on a 7‐month follow‐up CT scan (Figure 2H).

## 3 Discussion

In the present case, the geriatric patient exhibited profound surgical anxiety; accordingly, a percutaneous biopsy and CRA strategy were prioritised over thoracoscopic resection. Leveraging robotic navigation, we achieved single‐trajectory placement of the coaxial system and successfully executed transthoracic lung biopsy followed by CRA of the sub‐solid nodule. Histopathological assessment of the acquired cores demonstrated nonspecific inflammatory alterations, while complete on‐treatment ice‐ball coverage confirmed by immediate CT resulted in radiological eradication of the lesion and substantial amelioration of the patient's oncological anxiety.

Although there are diverse opinions regarding the choice between cold and heat, CRA has several advantages over radiofrequency ablation (RFA) and microwave ablation (MWA), including the ability to visualise the ice ball, minimal procedural pain and strong immunomodulatory effects [3]. However, multiple freehand needle manipulations to achieve a satisfactory cryoprobe position in conventional CRA may lead to unnecessary CT scans and complications. In addition, multiple cycles of freezing–thawing are necessary to achieve an effective ablation, resulting in a more time‐consuming treatment compared to RFA and MWA.

Robotic‐assisted system, as an innovative technique, offers high needle placement accuracy, reduces the number of manipulations and CT scans, and decreases the incidence of complications, meeting higher clinical treatment requirements for patients [4, 5]. In a comparative cohort study, it has been demonstrated that robotic‐assisted RFA of pulmonary metastases using general anaesthesia and high‐frequency jet ventilation is feasible and safe, with a 100% technical success rate, a lower incidence of adverse events and fewer manipulations than conventional freehand placement [4]. Considering the clinical characteristics and conditions of the patient, robotic‐assisted synchronous percutaneous lung biopsy and CRA were deemed optimal by the MDT because of enhanced precision, decreased radiation dose and fewer complications. To the best of our knowledge, this is among the first cases of a pulmonary sub‐solid nodule managed successfully with this integrated robotic approach.

The robotic‐assisted navigation system used in this study, which consists of an optical navigation system, a surgical planning system and a robotic arm placement and puncturing system, can offer several key advantages, such as the reconstruction of a visualised 3D model of various anatomical components, precise tracking of the actual position and orientation of the patient, comprehensive planning of trajectories, real‐time recording of respiratory motion and stable needle‐holding and guiding needle insertion [6]. These lead to more precise performance during the procedure, reducing unnecessary radiation exposure and lung injury resulting from repeated needle insertion attempts. A prospective clinical study has demonstrated that the one‐time success rate of percutaneous needle placement using the same robotic‐assisted system was 100% with significantly lower CT scans [6]. Consistent with the aforementioned features and findings, the puncture needle was successfully placed in the target lesion on the first attempt of this report, without requiring any adjustment to the position or angle of the needle, resulting in a reduction in the necessity for CT scans. Meanwhile, the procedure duration of 94 min in this case report was also shorter compared to a retrospective study with a mean procedure time of 103.24 ± 38.3 min by conventional CT‐guided manual CRA [7]. After proficiently mastering the robotic‐assisted system for lung biopsy and CRA, the procedure duration has gradually decreased and trended towards stability in some other cases, which will be reported in the future.

In summary, robotic‐assisted CT‐guided percutaneous lung biopsy and CRA are safe, feasible, less invasive and more accurate procedures to treat malignant pulmonary nodules, particularly for patients in whom surgery is contraindicated. It can maximise pulmonary function reserve compared with surgical resection and achieve satisfactory accuracy while also reducing lung injury, radiation dose and duration compared to traditional freehand manipulation. Further prospective investigations with long‐term follow‐up are warranted.

## Author Contributions

**Xiuping Wu:** data curation, formal analysis, writing – original draft. **Jielong Lin and Lianyue Yang:** methodology, investigation, resources. **Xin Wei and Haoran Xu:** validation, visualisation. **Yanwei Chen and Shi Yue Li:** conceptualization, supervision, project administration, writing – review and editing.

## Funding

The current report was sponsored by the Guangdong Provincial Science and Technology Program Project (grant number: 2025A0505010028).

## Consent

The authors declare that written informed consent was obtained for the publication of this manuscript and accompanying images using the consent form provided by the Journal.

## Conflicts of Interest

The authors declare no conflicts of interest.

## Data Availability

The data that support the findings of this study may be available upon reasonable request from the corresponding authors.
